# Efficacy of Anti-VEGF/VEGFR Agents on Animal Models of Endometriosis: A Systematic Review and Meta-Analysis

**DOI:** 10.1371/journal.pone.0166658

**Published:** 2016-11-17

**Authors:** Shuangge Liu, Xiaoyan Xin, Teng Hua, Rui Shi, Shuqi Chi, Zhishan Jin, Hongbo Wang

**Affiliations:** Department of Obstetrics and Gynecology, Union Hospital, Tongji Medical College, Huazhong University of Science and Technology, Wuhan, China; Yale University School of Medicine, UNITED STATES

## Abstract

**Background/Objective:**

Vascular endothelial growth factor (VEGF) is the most important promotor of angiogenesis. Some studies indicate that anti-angiogenic agents that interfere with VEGF and its receptor (VEGFR), i.e., anti-VEGF/VEGFR agents, may be applied to treat endometriosis. This meta-analysis investigated the efficacy of anti-VEGF/VEGFR agents in animal models of endometriosis.

**Methods:**

A systematic literature search was performed for animal studies published in English or Chinese from January 1995 to June 2016, which evaluated the effect of anti-VEGF/VEGFR agents on endometriosis. The databases were: PubMed, Web of Science, BIOSIS, Embase, and CNKI. The quality of included studies was assessed using the SYRCLE tool. The random-effect models were used to combine the results of selected studies. Heterogeneity was assessed using H^2^statistic and I^2^ statistic. Subgroup analyses were performed to determine the source of heterogeneity in endometriosis scores and follicle numbers.

**Results:**

We identified 13 studies that used anti-VEGF/VEGFR agents in various animal models. The meta-analysis showed that anti-VEGF/VEGFR agents were associated with smaller size (standardized mean difference (SMD) –0.96, 95% CI –1.31 to –0.62; *P* < 0.0001) and weight (SMD –1.70, 95% CI –2.75 to –0.65; *P* = 0.002) of endometriosis lesions, relative to the untreated controls, as well as a lower incidence rate of endometriosis (risk ratio 0.26, 95% CI 0.07 to 0.93; *P* = 0.038) and endometriosis score (SMD –1.17, 95% CI –1.65 to –0.69; *P* < 0.0001); the number of follicles were similar (SMD –0.78, 95% CI –1.65 to 0.09; *P* = 0.08).

**Conclusions:**

Anti-VEGF/VEGFR agents appeared to inhibit the growth of endometriosis, with no effect on ovarian function. Anti-angiogenic therapy may be a novel strategy in treating endometriosis.

## Introduction

Endometriosis is a common benign disease in women of reproductive age. The pathogenesis of endometriosis is not completely understood, but rates of recurrence at 2 and 5 years are ~21.5% and 40~50%, respectively [1]. Chronic pelvic pain and infertility are very common [2, 3], which markedly affect patients’ quality of life and increase the economic burden of the health-care system [4].

The current treatment of endometriosis involves surgical removal of the endometriotic lesions and pharmacological therapy. Pharmacological therapy mainly refers to suppression of endogenous estrogen synthesis with oral contraceptives, gonadotropin-releasing hormone (GnRH) agonists, aromatase inhibitors, and androgenic agents. However, the disease may recur after surgical excision, or after drug withdrawal, and the substantial side effects associated with this class of drugs limit their long-term use. Therefore, reliable new modalities for the long-term treatment of endometriosis are required.

It is widely accepted that angiogenesis is pivotal to the establishment of endometriosis lesions and their growth in ectopic sites [5]. Accordingly, anti-angiogenesis therapy may be an important approach in the management of endometriosis. Numerous studies have indicated that various anti-angiogenic agents may be promising candidates for endometriosis therapy, but there have been no clinical studies.

Angiogenesis is mainly mediated by vascular endothelial growth factor (VEGF) and its receptor (VEGFR). Efforts to suppress angiogenesis have targeted the VEGF/VEGFR pathway through anti-VEGF antibodies and VEGFR inhibitors [6]. The present meta-analysis systematically reviewed relevant studies of endometriosis therapies that applied either anti-VEGF antibodies or VEGFR inhibitors using animal models of the disease.

## Methods

### Literature search

We searched the following 5 online databases for papers published from January 1995 to June 2016: PubMed, Web of Science, BioSciences Information Service (BIOSIS) Previews, Embase, and Chinese National Knowledge Infrastructure (CNKI). We used combinations of the keywords “endometriosis”, “adenomyosis”, “endometrio*”, “angiogenesis inhibitors”, “angiogenesis inhibit*”, “vascular endothelial growth factors”, “antiangiogen*”, “anti-VEGF*”, “VEGF-target*”, “antibodies, monoclonal”, "protein-tyrosine kinases", “sorafenib”, “sunitinib”, “cediranib”, “vandetanib”, “bevacizumab”, “ranibizumab”, and “pazopanib”. Results were limited to animal studies. The search was limited to articles published in English or Chinese.

Abstracts were screened independently by 2 reviewers (Liu S and Xin X) to identify studies that met the inclusion criteria (below). The full search strategies are available in S1 File. The supporting PRISMA checklist is available in S1 Table.

### Inclusion and exclusion criteria

For inclusion in this meta-analysis, the selected studies included the following: angiogenesis inhibitors used as monotherapy; animal model of endometriosis; the number of animals per group was reported; outcomes were lesion size (volume or area) or lesion weight, rates of endometriosis incidence, or endometriosis score; and the full text was available. Experiments that used additional drugs as coordinated therapy were excluded. For studies in which there was disagreement between the 2 reviewers, consensus was met through discussion with a third reviewer (Hua T).

### Data extraction

The following data were extracted from the included studies: author; year; animal species; age; weight; experiment drug; control drug; animal number; type of animal model; time of experiment drug; administration route; dosage; and outcome measure. We extracted data regarding the outcome parameters (mean and standard deviation) from both the control and treatment groups to compare drug efficacy. When the outcome parameter was assessed with mean and standard error, we converted the standard error into standard deviation. When different angiogenesis inhibitors were assessed within multiple groups in one study, the data from each group were extracted as an individual experiment for analysis. When data were expressed serially at different time points, only the result of the final time point was included. When the outcome data was given only in graph format, we tried to contact the correspondence author of the article to ask for the original data, and if no reply was received, we used Engauge Digitizer software to measure graphically the data as presented. Two reviewers (Liu S and Xin X) independently extracted the data.

### Quality assessment

We assessed risk of bias among the studies using the protocol format of the Systematic Review Centre for Laboratory Animal Experimentation (SYRCLE) tool [7], which consists of the following questions: Was the allocation sequence adequately generated and applied?; Were the groups similar at baseline or were they adjusted for confounders in the analysis?; Was the allocation adequately concealed?; Were the animals randomly housed during the experiment?; Were the caregivers and investigators blinded to the intervention that each animal received?; Were animals selected at random for outcome assessment?; Was the outcome assessor blinded?; Were incomplete outcome data adequately addressed?; Are reports of the study free of selective outcome reporting?; and Was the study apparently free of other problems that could result in high risk of bias?

### Data analysis

The standardized mean difference (SMD) was the preferred statistic for pooling the continuous data outcome, and the risk ratio (RR) was preferred for the outcome of binary data. The 95% confidence intervals (CIs) of all results were calculated. Statistical significance was set at *P* < 0.05. Random effect models were used to calculate the pooled outcomes for it perform better than the fixed effect models with respect to coverage probabilities particular in small meta-analysis [8].

Statistical heterogeneity was assessed using the H^2^statistic and I^2^ statistic and its 95% CIs. H^2^ is the χ^2^ heterogeneity statistic divided by its degrees of freedom, and I^2^ is a transformation of H^2^ that describes as the proportion between studies variance that is due to inter-study heterogeneity rather than chance [9]. Heterogeneity was regarded as substantial if there was either I² more than 50% [9, 10], or H^2^ more than 1 [11]. When the results revealed statistically significant heterogeneity, possible explanations were investigated by subgroup analysis based on animal species, modelling, drug name, and drug administration. The effect of an individual study on the summary estimate was accessed using sensitivity analyses by excluding each study at a time from the meta-analysis. We assessed the presence of publication bias by using funnel plots [12], Begg’s test [13], Egger’s test [14] and Trim and Fill analysis [15] if there were more than 10 of the included experiments presented for the outcome measures. *P* < 0.10 was considered statistically significant. The data were analyzed using the statistical software package Stata version 12.0.

## Results

### Description of the included studies

**Fig 1:**
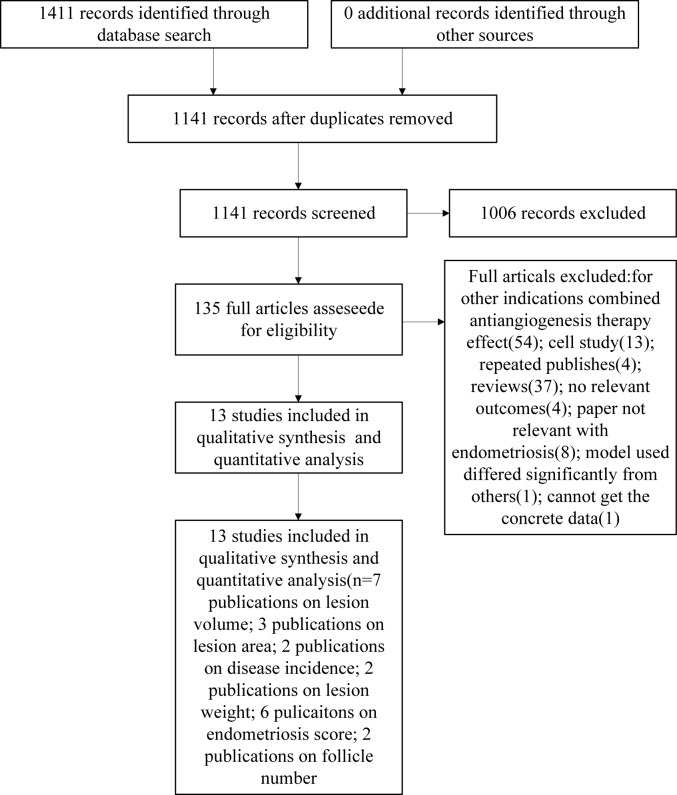
Flow chart of the study selection process.

We identified 1411 publications from the above-described electronic search, of which 270 were duplicates, and thus there were 1141 unique publications (Fig 1). After screening the titles and abstracts, 135 publications were found potentially relevant and the full texts were retrieved. We screened the 135 full publications and excluded an additional 122 publications for the following reasons: 54 combined anti-angiogenesis therapy effects; 13 were cell studies; 4 duplicate publications; 37 reviews; 4 with no relevant outcomes; 8 were not relevant to endometriosis; 1 model was significantly different from the other studies; and 1 had no obtainable concrete data. Finally, the present systematic review included 13 articles comprising 16 animal experiments with 245 animals, published between January 1995 and June 2016, which met the inclusion criteria [16–28] (Table 1). The characteristics among these studies varied considerably. The characteristics of animals themselves differed substantially between the studies. Eight of the studies used rat, two of the studies used nude mice, and others used SCID mice, BALB/c mice, Syrian golden hamsters and ovariectomized Rhesus monkeys. Among these studies, three of the studies used bevacizumab, three used sunitinib, three used sorafenib, two used anti-VEGF antibody(no specific name), while others used cediranib, pozopanib, ranizumab, SU5416 and anti-Flk1 antibody. Modelling methods between studies are different: seven studies used method of autotransplantation of uterine tissue to the peritoneum; one used transplantation of one of the uterine horns to the bowel mesentery; one used subcutaneous injection of human endometrium; one used abdominal subcutaneous injection of eutopic endometrium of human; one used induction menstrual bleeding mimic retrograde menstruation and one used deep nodules sutured onto the parietal peritoneum of the mice.

**Table 1:** Characteristics of the included animal studies.

Author, y	Species	Age	Weight	Animals ^a^	Animal model	Experimental drug	Control drug	Dosage, route	Treatment timing	Outcome measures
Abbas, 2013	Rats	NA	180–250 g	6/9	Autologous transplantation of endometrial onto the peritoneum	Sunitinib	Vehicle	10 mg/kg/d, IP	Begun 28 d after operation, for 21 d	Cyst cross-sectional area
Hull, 2003	Nude mice	5 wk	20–25 g	4/4	SC inject human endometrium	Antihuman VEGF antibody	Goat IgG	5 μg, SC	Immediately after tissue injections; for 10 d	No. of mice with lesions; No. of lesions in mice
Jiang, 2007	SCID mice	6–8 wk	20–25 g	10/10	Eutopic endometrium of human abdominal SC. Injection	Anti-VEGF antibody	PBS	3 mg/kg/d, IP	Begun 3rd wk post-operation, for 14 d	Lesion volume; lesion weight; MVD
LaSChke, 2006	Syrian golden hamsters	8–10 wk	60–80 g	5/8	Dorsal skinfold chamber	SU5416 ^b^	DMSO	25 mg/kg, IP	Day of operation, for 14 d	Lesion area; vascular area; MVD
Leconte, 2015	Nude mice	NA	NA	15/15	Deep nodules was sutured onto the parietal peritoneum of the mice	Sorafenib	PBS	50 mg/kg/d, oral	Begun day 7 post-operation; 5 d/wk for 2 wk	Tumor volume; pathological score; body weight
Liu, 2015	Sprague-Dawley rats	6–8 wk	NA	10/10	Surgically transplant autologous fragment onto the peritoneum	Cediranib	Saline + DMSO	4 mg/kg/d, IG	24 d after the operation, once daily, for 12 d	Lesion volumes; fibrosis; MVD; serum VEGF levels; proliferation; apoptosis; histology of ovary & uterus
Ozer, 2013a	Wistar Albino rats	5–6 wk	220–280 g	11/11	Autologous fragment of endometrial tissue onto the inner surface of the abdominal wall,	Bevacizumab	None	2.5 mg/kg, IP	Begun 3 d after the second operation & administered again after 10 d	Endometriosis score; histologic scores of VEGF & sFLT; number of primordial follicles
Ozer, 2013b	Wistar Albino rats	5–6 wk	220–280 g	11/11	Autologous fragment of endometrial tissue onto the inner surface of the abdominal wall	Sorafenib	None	30 mg/kg/d, gavage	Begun 3 d after 2nd operation & for 10 d	Endometriosis score; histologic scores of VEGF & sFLT; number of primordial follicles
Pala, 2015	Sprague–Dawley rats	NA	200–220 g	7/7	Autologous endometrium sutured to the left anterolateral peritoneal surface	Sunitinib	Tap water	3 mg/kg/d, oral	Begun 31 d after operation, lasted for 4 wk	Volume of lesion; histopathological score of implants; extent & severity of adhesions; plasma & peritoneal levels of VEGF,TNF-a, PTX3
Park, 2004	Ovariectomized Rhesus monkeys	NA	NA	6/6	Induce menstrual bleeding, then administer drugs. On day 7 of the menstrual cycle, the cul-de-sac was seeded with endometrial fragments to mimic retrograde menstruation.	Goat anti-Flk1 antibody	Non-specific IgG	0.07 mg/kg, NA	NA	Number of mice develop lesion
Ricci, 2011	BALB/c mice	2 mo	NA	12/12	Transplantation of one of the uterine horns to the bowel mesentery	Bevacizumab	Saline	5 mg/kg, IP	Post-surgery days 15, 18, 21, 24, & 27	Lesion number. lesion volume; MVD; proliferation; apoptosis;
Sevket, 2013	Wistar albino rats	10–12 wk	200–250 g	9/9	Autologous fragment of endometrial tissue onto the inner surface of the abdominal wall	Ranibizumab	None	0.6 mg/kg, IP	Day 1 & 14 after second operation, observe 28 d	Lesion volume; lesion weight; histologic score
Soysal, 2014	Wistar rats	8 wk	200–250 g	10/10	Surgically transplant autologous endometrium fragment onto the peritoneum	Bevacizumab	Saline	2.5 mg/kg, single IP	3 wk after operation, single IP	Lesion area; adhesion score; endometrial score; pro-apoptotic & anti-apoptotic gene expression
Yildiz, 2015a	Wistar-Albino rats	NA	220–240 g	8/8	Autotransplantation of uterine tissue to the peritoneum	Pazopanib	NS	80 mg/kg, oral	Begun 21 d after operation, for 14 d	Endometriosis score; ovarian follicle number; staining scores of VEGF, CD 117 & Bax
Yildiz, 2015b	Wistar-Albino rats	NA	220–240 g	8/8	Autotransplantation of uterine tissue to the peritoneum	Sunitinib	NS	10 mg/mL, oral	Begun 21 d after operation, for 14 d	Endometriosis score; ovarian follicle number; staining scores of VEGF, CD 117 & Bax
Yildiz, 2015c	Wistar-Albino rats	NA	220–240 g	8/8	Autotransplantation of uterine tissue to the peritoneum	Sorafenib	NS	30 mg/d, IP	Begun 21 d after operation, for 14 d	Endometriosis score; ovarian follicle number; staining scores of VEGF, CD 117 & Bax

^a^ *n* Control/*n* experimental groups

^b^ VEGF inhibito

SC, subcutaneous; IG, intragastric; NA, not available or not mentioned; NS, normal saline; PBS, phosphate-buffered saline; DMSO, dimethyl sulfoxide; MVD, microvessel density; SC, subcutaneous; sFLT, soluble fms-like tyrosine kinase receptor; TNF-a, tumor necrosis factor-alpha; PTX3, pentraxin 3; Bax, BCL2 associated X, apoptosis regulator.

### Effect of interventions

#### Lesion size

**Fig 2:**
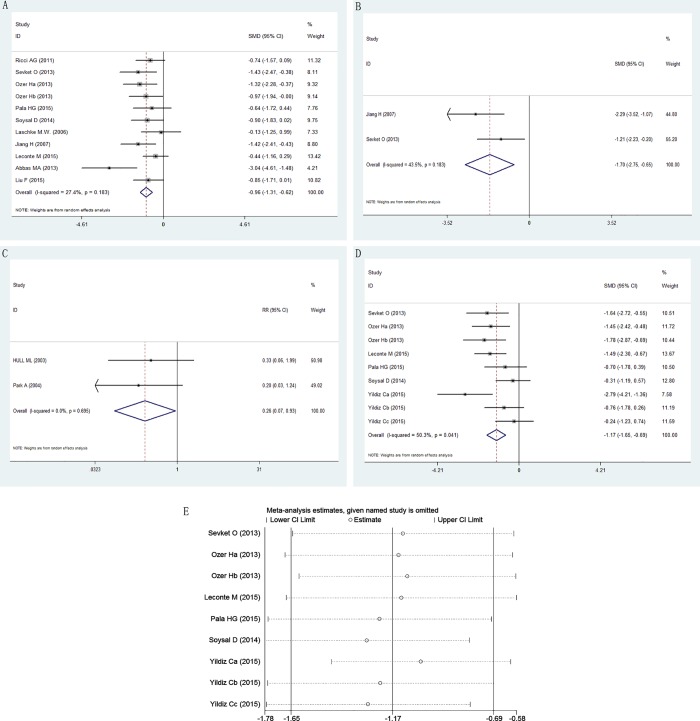
Effect of anti-VEGF/VEGFR agents on endometriosis lesion growth. Effect on (A) lesion size; (B) lesion weight; (C) endometriosis incidence; (D) endometriosis score; (E) sensitivity analysis for the outcome of endometriosis score.

Eleven of the 16 experiments reported outcomes related to endometriosis size (volume or area) [16, 18–23, 25–27]. Seven different anti-VEGF/VEGFR agents were used, if it is considered that Jiang [11] used a same drug from the others, there were six. Taken together, these experiments indicated that anti-VEGF/VEGFR agents were associated with significant inhibition of the growth of endometriosis lesions (SMD –0.96, 95% CI –1.31 to –0.62; *P* < 0.0001; Fig 2A) with a low heterogeneity in the estimates (I^2^ = 27.4%, 95% CI 0% to 64%; H^2^ = 0.38).

#### Lesion weight

The effect of anti-VEGF/VEGFR agents on the weight of endometriosis lesions in animal models was evaluated. Only 2 experiments reported this outcome [18, 26]. The mean weight of endometriosis lesions in the groups treated with anti-VEGF/VEGFR agents was significantly less than that of the untreated animals (SMD –1.70, 95% CI –2.75 to –0.65; *P* = 0.002; Fig 2B). The heterogeneity was moderate (I^2^ = 43.5%, H^2^ = 0.77).

#### Endometriosis incidence

Two experiments reported outcomes related to the presence of endometriosis [17, 24]. The incidence rate of endometriosis in the treatment group was significantly less than that of the control (RR 0.26, 95% CI 0.07 to 0.93; *P* = 0.038; Fig 2C). There was no obvious heterogeneity (I^2^ = 0%, H^2^ = 0). Considering the low number of animal studies included in each outcome measure, these results must be interpreted with caution.

#### Endometriosis score

Endometriosis score outcomes after anti-VEGF/VEGFR therapy were reported in 9 experiments [20, 22, 23, 26–28]. Taken together, these experiments showed that anti-angiogenesis intervention was associated with a lower endometriosis score (SMD –1.17, 95% CI –1.65 to –0.69; *P* < 0.0001) with a large heterogeneity in the estimates (I^2^ = 50.3%, 95% CI 0% to 77%; H^2^ = 1.01; Fig 2D).

To determine whether the different effects were caused by different scales of measurement, heterogeneity was investigated after subgroup analysis (Table 2). The analysis showed that anti-VEGF/VEGFR agents had a significant inhibitory effect on endometriosis score in both rats (SMD –1.13, 95% CI –1.68 to –0.57; *P* < 0.0001) and mice (SMD –1.49, 95% CI –2.30 to –0.67; *P* < 0.001). Subgroup analysis of animal species did not reduce heterogeneity.

**Table 2:** Subgroup analysis of the 16 included experiments for the effect of anti-VEGF/VEGFR agents on endometriosis score.

						Heterogeneity		Effect size	
		SMD	LL	HL	n	I^2^	95%CI	Z	P
Overall		–1.170	–1.651	–0.688	9	50.3%	0%~77%	4.76	<0.0001
Species	Rat	–1.127	–1.675	–0.579	8	53.9%	0%~79%	4.03	<0.0001
	Mouse	–1.488	–2.303	–0.673	1	—	—	3.58	<0.0001
Modelling	Autotransplantation	–1.127	–1.675	–0.579	8	53.9%	0%~79%	4.03	<0.0001
	Deep nodule implantation	–1.488	–2.303	–0.673	1	—	—	3.58	0.0001
Drug administration	IP	–0.880	–1.591	–0.169	4	52.8%	0%~84%	2.43	0.015
	Oral	–1.420	–2.059	–0.781	5	45.0%	0%~80%	4.35	<0.0001
Drug name	Ranibizumab	–1.636	–2.720	–0.552	1	—	—	2.96	0.003
	Bevacizumab	–0.861	–1.973	0.251	2	65.2%	—	1.52	0.129
	Sorafenib	–1.170	–2.062	–0.279	3	61.5%	0%~89%	2.57	0.011
	Sunitinib	–0.733	–1.476	0.010	2	0.0%	—	1.93	0.053
	Pazopanib	–2.787	–4.210	–1.364	1	—	—	3.84	<0.0001

SMD, standardized mean difference; LL, lower limit; HL, higher limit; 95%CI, 95% confidence interval.

We also conducted a subgroup analysis of the administration route of the drug (Table 2). We found that neither intraperitoneal injection (IP, SMD –0.88, 95% CI –1.59 to –0.17; *P* = 0.01) nor oral administration (SMD –1.42, 95% CI –2.06 to –0.78; *P* < 0.0001) influenced the therapeutic effect of the agents. Subgroup analysis of agents delivered orally slightly reduced heterogeneity (I^2^ = 45.0%), while the subgroup analysis based on IP injection did not change the high heterogeneity level (I^2^ = 52.8%). We also conducted a subgroup analysis of modeling and drug name (Table 2).

We also conducted a sensitivity analysis (Fig 2E). We found that the result of Yildiz Ca et al. [28] was more positive than others, and heterogeneity decreased (I^2^ = 33.8%) after removal of this study. However, it cannot be excluded because we did not find any reason to do that after exploring clinical heterogeneity and methodological heterogeneity. The difference between this study and the others is the drug been used, perhaps pazopanib is more effective in treating endometriosis. This needs further study to confirm.

### Effect of anti-VEGF/VEGFR agents on ovarian function

**Fig 3:**
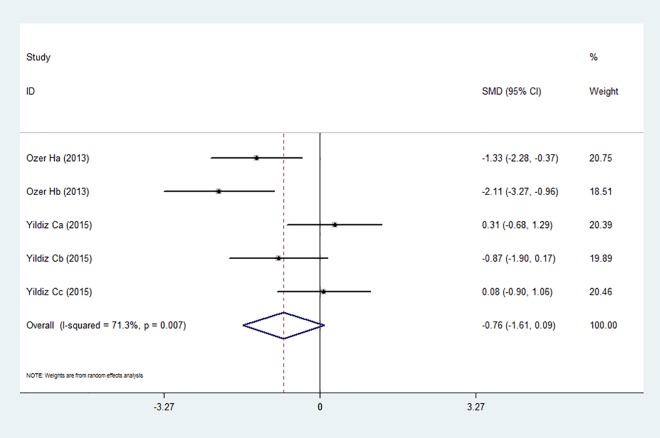
Effect of anti-VEGF/VEGFR on follicle number.

We also analyzed the influence of anti-VEGF/VEGFR therapy on ovarian function. We chose the number of follicles as the outcome measure. There were 5 experiments that reported the number of follicle alteration after administrating the agents [22, 28]. Taken together, angiogenesis inhibition therapy had no obvious effect on follicle number (SMD –0.78, 95% CI –1.65 to 0.09; *P* = 0.08) and the heterogeneity was high (I^2^ = 71.3%, 95% CI 27% to 89%; H^2^ = 2.49; Fig 3). Subgroup analysis showed that the follicular count method may account for the source of heterogeneity. In the Ozer at al. [22] study, the number of primordial follicles was determined (I^2^ = 4.9%) while in Yildiz at al. [28], the numbers of primordial, primary and secondary, and antral follicles were assessed (I^2^ = 30.7%).

### Publication bias

We conducted publication bias test for the outcome of lesion size. *P* value for Begg’s test is 0.062 (continuity corrected), and for Egger’s test is 0.019(S1 Fig), indicating a potential existence of publication bias across included studies. So we conducted trim and fill approach, result showed not any theoretically missing studies was found, indicating the outcome was stable. For other outcome measure, we did not take the publication bias test because their included experiments were less than 10.

### Risk of bias and quality of included studies

**Fig 4:**
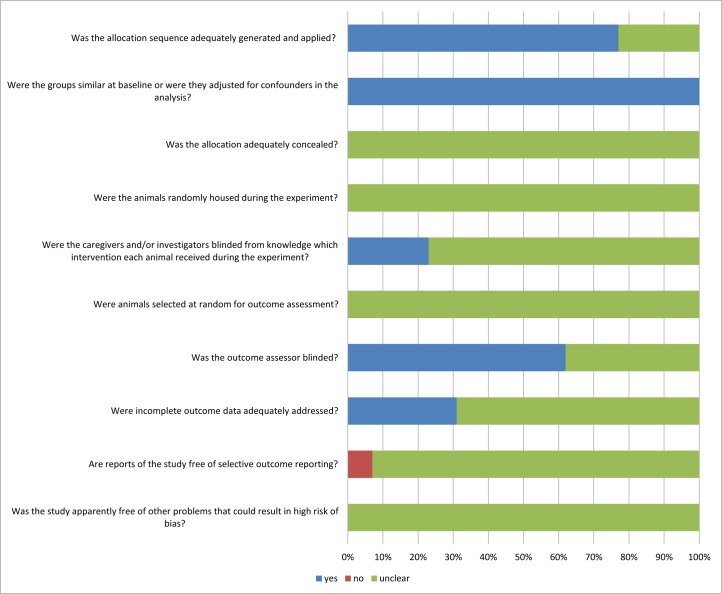
SYRCLE’s Risk of bias: Yes = low risk of bias, no = high risk bias, unclear = unclear risk of bias.

The quality of the 13 studies included in this systematic review was assessed using the SYRCLE tool (Fig 4). Ten (77%) of the studies stated that the allocation was randomized, but only one study stated the method of randomization. No study described whether the allocation was adequately concealed. Most of the domains were poorly described, which indicates that these animal studies were at high risk of bias.

## Discussion

### Summary of the evidence

Medical treatments intended to reduce endogenous estrogen production is the conventional treatment for endometriosis in clinical practice. Although they are effective in suppressing the growth of endometriosis lesions and reducing pain, the adverse effects limit their long-term use. VEGF is involved in the pathogenesis of endometriosis. Therefore, suppression of vascular development by inhibiting VEGF or its receptor may be a novel therapeutic strategy for the treatment of endometriosis [24, 29, 30].

The present study assessed the preclinical literature reporting administration of VEGF/VEGFR angiogenesis inhibitors in the treatment of endometriosis. The meta-analysis showed that anti-VEGF/VEGFR treatment had a significant inhibitory effect on endometriosis size (SMD –0.96, 95% CI –1.31 to –0.62), reduction of endometriosis weight (SMD –1.70, 95% CI –2.75 to –0.65), and endometriosis score (SMD –1.17, 95% CI –1.65 to –0.69) when compared to control animals. In addition, there is evidence that anti-VEGF/VEGFR therapy can reduce the incidence of endometriosis by 74%. Considering the low number of animal studies included in each outcome measure, these results must be interpreted with caution.

The observed heterogeneity among the studies regarding endometriosis scores cannot be completely explained. We performed a stratified analysis of the animal species, route of drug administration, modeling method, and drug name, but no obvious source of heterogeneity was found. The sensitivity analysis showed that the study of Yildiz Ca et al. [28] may be the source of heterogeneity. However, it cannot be excluded because we did not find any reason to do that after exploring clinical heterogeneity and methodological heterogeneity. The difference between this study and the others is the drug used; pazopanib may be more effective in treating endometriosis, but why pazopanib is more effective than other drugs requires further studied. For the little number of included studies, we did not conduct meta-regression. In summary, it can be concluded that anti-VEGF/VEGFR agents may be beneficial in the treatment of endometriosis.

VEGF, as a key mediator of angiogenesis, its biological effects are primarily mediated through 2 high-affinity receptors on the surface of microvascular endothelial cells, i.e., VEGFR-1 and VEGFR-2. Hull et al. [17] was the first to report that treatment with a soluble truncated fms-like tyrosine kinase receptor 1 (Flt-1) or affinity-purified VEGF-antibody could significantly inhibit the growth of endometriotic lesions in nude mice by disrupting their immature microvasculature. Blocking VEGF to treat endometriosis decreases vascular density and cell proliferation and increases cell apoptosis, and reduces VEGF levels in the peritoneal fluid of the animals [31]. Vascular inhibitors can also inhibit angiogenesis via inhibition of the mitogen-activated protein kinase (MAPK) pathway [20]. Studies show that anti-angiogenic treatment can decrease new blood vessel formation, but cannot destroy old or stabilized vessels [32]. Agents should be given in the proliferative phase of the cycle, because it less effective in reducing lesion formation when endometrial tissue explants were exposed to progesterone [17] or during estrus [24]. Several studies have assessed the effect of other types of drugs that suppress angiogenesis in endometriosis with different results. These drugs included endostatin [29, 30, 33, 34], cabergoline [35, 36], and anginex [29].

The emerging interest in angiogenesis inhibitors is raising concerns about the safety of their long-term use. There is a potential adverse risk to important physiological functions regulated by VEGF or VEGFR, such as wound healing, hypertension, myocardial or peripheral ischemia [2, 37]. Most of these are reversible. However, for women with endometriosis, the effect on reproductive function is of concern. Becker et al. [38] showed that treatment of endometriosis with anti-angiogenic agents did not affect reproductive functions such as the estrous cycle, corpus luteum formation, pregnancy, number of pups, or fetal vitality. In the included studies, Ozer et al. [22] reported that bevacizumab had no effect on ovarian reserve, but sorafenib adversely affected the ovarian reserve. However, another study found no adverse effects associated with sorafenib, sunitinib, or pazopanib therapy on ovarian follicle number [28]. Angiogenesis inhibition therapy cannot only disrupt the vascular supply, but can decrease the formation of adhesions [16]. Therefore, it can be assumed that anti-angiogenic agents may be useful in the treatment of infertility [39]. Pala et al. [23] found that sunitinib diminished the mean pain score, and no adverse side effects were observed. In the present study, our results show that angiogenesis inhibitors had no significant adverse effect on ovarian function. This result had a high level of statistical heterogeneity, which may be a reflection of different methodological design. In the study of Ozer et al. [22], primordial follicles were identified. However, the presence of primordial, primary and secondary, and antral follicles was assessed in the study of Yildiz at al. [28]. The adverse events associated with angiogenesis inhibitors in our meta-analysis include weight loss [20]. Weight loss may also be associated with anorexia and reduced food intake [40].

By using the SYRCLE’s tool for assessing risk of bias in animal studies, we found out that the overall reporting quality of the included studies is poor, since the methodology of most studies is unclear. The application of SYRCLE is very important in systematic reviews of animal studies, as it can reveal the source of heterogeneity, to some extent. There were only 13 studies included in this review. Such a small number of studies, and the uncertainty of methodological quality, may lead to over or underestimation of the effect of treatments on angiogenesis inhibition in endometriosis.

### Limitations

The present study provides a useful summary of the preclinical data of anti-VEGF/VEGFR angiogenesis inhibitors on endometriosis. However, there are several limitations to our approach. First, each dataset in our defined outcome variables was relatively small. Our search strategy was restricted to English and Chinese, and there are some missing studies. If the missing studies are taken into account, the estimates of the effect are likely overstated. In addition, though the estimates of heterogeneity is zero or very low in some of the outcomes, heterogeneity should also be a concern since it is very likely present but undetected (or underestimated) especially in small meta-analyses[41]. On the other hand, we conducted a test for publication bias for outcome of the lesion size, the results of Begg's test and Egger's test indicate a potential existence of publication bias, but no study was trimmed or filled. The reasons of the inconsistent results might be derived from small sizes of this study or the amount of included studies.

There were also methodological differences among the studies. The studies included in this review administered the anti-VEGF/VEGFR drug at different times relative to modeling (e.g., from the day of modeling to 31 days after modeling), and for different treatment period (3 days to 4 weeks) without a long-term follow-up. We know that endometriosis is a chronic disease that requires long-term therapy, and rates of relapse are high in a high estrogen environment. Therefore, we do not know the long-term effect of this class of drugs on endometriosis.

Another limitation of this meta-analysis is that combining data from different studies may hide slight variations that are relevant to drug efficacy. For example, we combined the results of different drugs within the predefined outcome measures, as there were too few experiments to assess them separately. This may increase the heterogeneity of outcomes.

Finally, the overall quality of the included studies constituted a high degree of inter-study heterogeneity, and a correlation between overall quality and drug effect was not clear. Measures to reduce bias need to be improved, such as randomization and blinding of investigators.

## Conclusions

Our meta-analysis shows that the use of anti-VEGF/VEGFR agents has a potential beneficial effect in the treatment of endometriosis. Since currently there have been no clinical investigations of the therapeutic effects of angiogenesis inhibitors in endometriosis, the results of this meta-analysis could provide an important reference for future preclinical animal trials or clinical trials.

## Supporting Information

S1 Fig Begg’s funnel plot overseeing publication bias of included studies for the outcome of lesion size. (TIF)

S1 File Search Strategy. (DOCX)

S1 Table PRISMA check list. (DOC)
