# Supplementary figures and images for: Efficacy of Anti-VEGF/VEGFR Agents on Animal Models of Endometriosis: A Systematic Review and Meta-Analysis
Source: PLoS One. 2016 Nov 17;11(11):e0166658. doi: 10.1371/journal.pone.0166658 (PMC5113963; doi:10.1371/journal.pone.0166658)

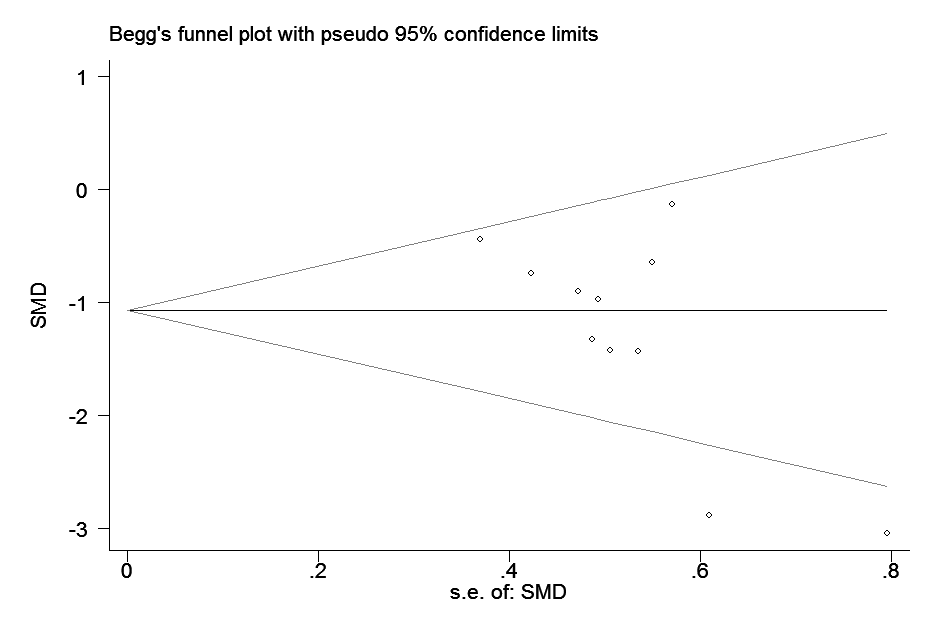

Supplement: S1 Fig — (TIF) [file pone.0166658.s001.tif]
