# Supplementary material for: Efficacy of Anti-VEGF/VEGFR Agents on Animal Models of Endometriosis: A Systematic Review and Meta-Analysis
Source: PLoS One. 2016 Nov 17;11(11):e0166658. doi: 10.1371/journal.pone.0166658 (PMC5113963; doi:10.1371/journal.pone.0166658)
Supplement: S1 File — (DOCX) [file pone.0166658.s002.docx]

**S1 File**

**Pubmed search strategy**

Search ((((("Endometriosis"[Mesh]) OR endometriosis[Title/Abstract]) OR adenomyosis[Text Word]) OR endometrio*[Text Word])) AND ((((((((((((("Angiogenesis Inhibitors"[Mesh]) OR "Vascular Endothelial Growth Factors"[Mesh]) OR vascular endothelial growth factor*[Text Word]) OR angiogenesis inhibit*[Text Word]) OR ((antiangiogen*[Text Word] OR anti-angiogen*[Text Word]))) OR anti-VEGF*[Text Word]) OR VEGF-target*[Text Word]) OR angiostatic agent*[Text Word]) OR "Antibodies, Monoclonal"[Mesh]) OR monoclonal antibodies[Text Word]) OR "Protein-Tyrosine Kinases"[Mesh]) OR tyrosine kinase inhibit*[Text Word]) OR (((((((sorafenib[Text Word]) OR sunitinib[Text Word]) OR cediranib[Text Word]) OR vandetanib[Text Word]) OR bevacizumab[Text Word]) OR ranibizumab[Text Word]) OR pazopanib[Text Word])) Filters: Journal Article; Publication date from 1995/01/01 to 2016/06/01; English

**Embase search strategy**

1. exp endometriosis/

2. endometriosis.ti,ab.

3. endometrio$.tw.

4. adenomyosis.tw.

5. 1 or 2 or 3 or 4

6. exp angiogenesis inhibitor/

7. exp vasculotropin/

8. vascular endothelial growth factor$.tw.

9. (angiogenesis adj5 inhibit$).tw.

10. (anti adj2 VEGF$).tw.

11. (anti adj1 angiogen$).tw.

12. VEGF TRAP$.tw.

13. VEGF-target$.tw.

14. agiostatic agent$.tw.

15. exp monoclonal antibody/

16. monoclonal antibodies.tw.

17. exp protein tyrosine kinase/

18. (protein tyrosine adj5 inhibit$).tw.

19. (sorafenib or sunitinib or cediranib or vandetanib or bevacizumab or ranibizumab or pazopanib).tw.

20. 6 or 7 or 8 or 9 or 10 or 11 or 12 or 13 or 14 or 15 or 16 or 17 or 18 or 19

21. 5 and 20

22. Limit 21 to (English language and exclude Medline journals and yr ="1995 -Current" and article)
